# Sunitinib treatment promotes metastasis of drug-resistant renal cell carcinoma via TFE3 signaling pathway

**DOI:** 10.1038/s41419-021-03511-3

**Published:** 2021-02-26

**Authors:** Luchao Li, Shuo Zhao, Zhengfang Liu, Nianzhao Zhang, Shuo Pang, Jikai Liu, Cheng Liu, Yidong Fan

**Affiliations:** 1 Department of Urology, Qilu Hospital, Shandong University, Jinan, China; 2 Department of Urology, Peking University Third Hospital, Beijing, China

**Keywords:** Renal cell carcinoma, Cell invasion, Prognostic markers

## Abstract

Receptor tyrosine kinase (RTK) inhibitors, such as sunitinib and sorafenib, remain the first-line drugs for the treatment of mRCC. Acquired drug resistance and metastasis are the main causes of treatment failure. However, in the case of metastasis Renal Cell Cancer (mRCC), which showed a good response to sunitinib, we found that long-term treatment with sunitinib could promote lysosome biosynthesis and exocytosis, thereby triggering the metastasis of RCC. By constructing sunitinib-resistant cell lines in vivo, we confirmed that TFE3 plays a key role in the acquired resistance to sunitinib in RCC. Under the stimulation of sunitinib, TFE3 continued to enter the nucleus, promoting the expression of endoplasmic reticulum (ER) protein E-Syt1. E-Syt1 and the lysosomal membrane protein Syt7 form a heterodimer, which induces ER fragmentation, Ca2^+^ release, and lysosomal exocytosis. Lysosomal exocytosis has two functions: pumping sunitinib out from the cytoplasm, which promotes resistance to sunitinib in RCC, releasing cathepsin B (CTSB) into the extracellular matrix (ECM), which can degrade the ECM to enhance the invasion and metastasis ability of RCC. Our study found that although sunitinib is an effective drug for the treatment of mRCC, once RCC has acquired resistance to sunitinib, sunitinib treatment will promote metastasis.

## Introduction

Renal cell carcinoma (RCC) accounts for approximately 2% of all adult malignancies worldwide^1^. Clear cell renal cell carcinoma (ccRCC), the most common histologic subtype of RCC^2^, is characterized by a loss of the von Hippel-Lindau (VHL) gene with induction of hypoxia-inducible factor (HIF) and vascular endothelium growth factor (VEGF)^3–6^. Radical nephrectomy can enable patients with early-stage RCC to obtain a very impressive 5-year survival rate (92.6%)^7^. However, over 20% of patients are primarily diagnosed at the metastatic stage, and over 30% suffer metastasis after surgery^8^. Anti-angiogenic receptor tyrosine kinase inhibitors (TKIs) such as sunitinib and sorafenib remain the first-line treatment for mRCC. Unfortunately, most patients eventually develop resistance to TKI treatment. For the clinical management of mRCC patients, a more definite understanding of the mechanisms of tumor metastasis and TKI resistance is urgently needed. Lysosomes are intracellular vesicles bound by phospholipid bilayer membranes. Their biological functions include clearance of dysfunctional organelles, degraded proteins, and phagocytosis^9–13^. The main lysosomal acid hydrolases, cathepsins (CTS), are involved in tumor progression 0tumor metastasis is the degradation of the extracellular matrix (ECM) by matrix metalloproteinases such as MMP9. The exocytosis of cathepsin B into the ECM via lysosomes can trigger MMP-9 activation so as to degrade the ECM and dissolute cell-cell adhesion molecules to promote cancer metastasis^14,15^. In addition, as a kind of hydrophobic weak base synthetic drug, sunitinib can be trapped and sequestered in acidic lysosomes so as to induce sunitinib resistance in ccRCC^16–18^. The coordinated lysosomal expression and regulation (CLEAR) gene network is the upstream regulator of lysosome biogenesis. Transcription factor EB (TFEB) and transcription factor E3 (TFE3) are two core activators of the CLEAR pathway and depend on mitochondrial translational initiation factor (MITF) family members^19–21^.

We treated a mRCC patient with sunitinib. After 4 years of continuous sunitinib treatment, the patient developed new metastasis to the cervical lymph nodes. Tandem mass tag (TMT) proteomic sequencing was used to analyze metastatic RCC lesions compared with sunitinib-sensitive RCC lesions. Surprisingly, we found that the lysosome enzyme pathway was strongly enriched in the up-regulated proteins, and the ECM receptor and cell adhesion pathways were enriched among the downregulated proteins. Based on the patient’s medication history, it is likely that the amplification of the lysosomal pathway is caused by TFEB/TFE3 translocation into the nucleus, thereby activating the CLEAR gene regulation network. We further infer that the new metastasis in the patient is most likely due to the fact that after drug resistance in the primary lesion, sustained TKI drugs still inhibited the VEGF-mTOR pathway, causing TFE3/TFEB to lose the restriction of phosphorylation and thus enter the nucleus. In other words, if the treatment strategy is not changed after primary lesion resistance to TKI, continuous TKI treatment will result in the activation of the TFEB/TFE3-lysosome pathway to trigger RCC metastasis.

## Materials and methods

### The iTRAQ quantitative proteomics

#### Protein extraction and labeling

Samples were obtained with the consent of the ethics committee of Qilu hospital and the patient herself. After treated with 1 ml of lysate containing 7 m urea, 2 m thiourea, and 0.1% chaps, tissues from each group were homogenized by TiO_2_ redox beads (70 Hz, 120 s at 5000 × *g* × 5 min at 4 °C). Collect the supernatant with centrifugal force of 15000 × *g* at 4 °C for 15 min. The protein concentration was determined according to the manufacturer’s protocol (Qinglian Biotech Co., Ltd, Beijing, China). In total 200 μg samples were digested overnight with 4 μg trypsin at 37 °C. labeling with incubating 200 μg protein with a reducing agent (5 μl, 200 mm) at 55 °C for 60 min, then adding iodoacetamide (5 μl, 375 mM) for 10 min without light at RT. Next, adding dissolution buffer (200 μl 100 mm Absciex), and centrifuged at 15000 × *g* for 15 min. Digesting each sample with trypsin (4 μg) overnight at RT. Then lyophilizing all the samples and dissolving with dissolution buffer (100 mM). Labeling samples according to the protocol of TMT kit (PN: 90064, Thermo Scientific, Waltham, MA USA).

### Identification of peptide

Resuspending the peptide by buffer A (0.1% FA, 2% ACN, 20 μl) and collecting the supernatant with centrifugal force of 12000 rpm for 10 min. Then a nano UPLC-MS/MS system which was composed of a Nanoflow HPLC system (EASY-nLC1000 system, Thermo Scientific) and Q-Exactive mass spectrometer (Thermo Scientific) was used to identify. The database is NCBI Mus musculus_20160428 and Proteome Discoverer 1.4 is used for data processing.

#### Protein identification and quantification

Parameters for identifications: precursor ion mass tolerance, ±15ppm; fragmentation mass tolerance, ±20mmu; max missed cleavages,2; static modification, carboxyamidomethylation (57.021 Da) of Cys residues; dynamic modifications, oxidation modification (+15.995 Da) of Met residues *p* ≤ 0.05 and difference ratio≥1.2 were chosen for bioinformatics analysis.

### **C**ell culture

The HeLa cell line and 786 O cell line were obtained from the Chinese Academy of Science Cell Bank (Shanghai, China). The media of culturing 786 O, 786 O/OE, 786 O/OE-SR cells were RPMI-1640 supplemented with 10% fetal bovine serum, 100 units/ml penicillin, and streptomycin (Gibco Invitrogen, Monza, Italy). In total 293 T was cultured in Dulbecco’s modified Eagle’s medium (DMEM, Gibco Invitrogen) with 10% FBS, 100 units/ml penicillin, and streptomycin. All cells were maintained under the condition of 37 °C with 5% CO_2_. All the cell lines were recently authenticated STR without mycoplasma contamination.

### Reagents and antibodies

Sunitinib (s7781), DC661(S8808), MG132(S2619), 3-MA(S2767), CA-074ME(S7420), Ionomycin (s7074), Puromycin(s7417) were purchased from Selleckchem (Houston, TX, USA). Fluo-3(46393), Earle’s Balanced Salt Solution (EBSS, E7510) were purchased from Sigma-Aldrich. The primary antibodies against E-Syt1(ab118805), FTH1(ab231253), TFE3 (ab196681), CTSB (ab125067), Syt7(121383), mCherry (ab183628), GAPDH (ab8245), LC3B (ab192890), GFP (ab290), HA (ab9110), mTOR (ab32028), p-mTOR (ab109268), MMP9 (ab76003), H3 (ab1791) were purchased from Abcam (Cambridge, Cambridgeshire, UK). The primary antibody of FAM14B (21573-1-AP) were purchased from Proteintech (Chicago, IL, USA). The horseradish peroxidase (HRP) labeled Goat anti-Rabbit (ab6721) and Rabbit anti-Mouse (ab6728) secondary antibodies were purchases from Abcam.

### Cell viability assay

In total 3 × 10^3 cells/well were seeded into 96-well plates overnight. Then the cells were treated with new complete media containing sunitinib with different concentration for 48 h. After that, each well was added 10 µL Cell Counting Kit-8 (CCK-8) solution (Dojindo, Kumamoto, Japan) and the plates were incubated at 37 °C for 1 h. Finally, a microplate reader was used to test the absorbance at 450 nm. Three separated repeats were performed.

### Wound healing assay

The indicated cells were plated in six-well plates to test the migration capability. Drawing a line shape in the wells of the 6-well by using a 100-μL pipette tip. Then replace the medium with 2 mL of serum-free culture medium containing different concentration of sunitinib. Scratches were pictured at 0 h and 24 h after the medium was replaced immediately after. Changes in the scratches were imaged after 24 h. The cells migration capability was judged based on variations in the widths of scratches. Three separated repeats were performed.

### Transwell assay

Before seeding 2 × 10^^4^ cells (200 μL serum-free RPMI-1640 medium) into the champers (Millipore, Billerica, MA, USA), the upper facial of membrane (8 μm porefilters) was coated with the 50 μL matrigel (200 ng/mL, BD Biosciences, NJ, USA) and RPMI-1640 with 10% FBS was added to the lower chambers. Then the seeding cells were incubated 12 h for migration and 24 h for invasion at 37 °C. Cells retained on the upper facial of the membrane were gently wiped off and cells migrated to the bottom of the membrane were fixed with 4% paraformaldehyde, stained by crystal violet solution for 20 min. Visualization and statistics under 100 times magnification. Experiments were repeated three times with similar results.

### Enzyme linked immunosorbent assay (ELISA)

ELISA for node mouse serum Ferritin (ab157713, Abcam) was performed according to the manufacturer’s instructions.

### Small interfering RNA (siRNA), plasmid, lentiviral synthesis

Small interference RNA (siRNA) targeting E-Syt1, TFE3, short hairpin RNA (shRNA) targeting E-Syt1, CTSB, FAM134B, Syt7, TFE3, overexpressing plasmids driving TFE3, E-Syt1, E-Syt1-GFP, FAM134B-mCherry, Sty7, Syt-GFP were synthesized by Genepharma (Shanghai, China). The lentivirus containing TFE3 overexpressing plasmid, sh-TFE3 plasmid, sh-Syt7 plasmid, EATR system, CCER system were synthesized by Shanghai Genechem Co., Ltd. Sty7-mCherry overexpressing plasmid and corresponding mutation plasmids were designed by Genepharma (Shanghai, China).

### Mass spectrum

A total of 786 O/OE-SR cells transfected with Esyt1-GFP or Syt-GFP plasmid, subjected to incubation with anti-GFP antibody. The proteins having interaction with Esyt1-GFP or Syt7-GFP were pulled down by Co-IP according to Kit’ protocols from TaKaRa (TaKaRa BIO, Dalian, China) and performed with MS analysis. SDS-PAGE gel was used to separate the peptide. We minced the gel and collected it in a test tube and dried it naturally. The peptide segments in the gel fragments were reduced by 10 mM DTT at 56 °C for 30 min, and then alkylated with 50 mM IAM for 30 min in the dark. Samples were digested by trypsin for 14 h at 37 °C. The samples were acidified by formic acid and desalted by C18 cartridge. Then samples were dried by vacuum centrifugation. The Orbitrap Q Exactive HF-X mass spectrometer (Thermo Fisher) was used to test samples.

### Immunohistochemistry

The clinical patient’ tumor tissues and xenograft tumor tissues were fixed in 10% formalin for 48 h. After dehydration and embedment, the tissues were made into paraffin sections. Then xylene and a series of descending dilutions of ethanol were used to deparaffinize. We used a microwave oven to perform antigen retrieval by heating to boiling in citrate antigen retrieval solution for 15 min. The activity of endogenous peroxidase was blocked by 0.3% Hydrogen Peroxide at room temperature for 20 min. The slides were then tested with the antibody of TFE3 respectively (1:200) at 4 °C overnight and then the sections were tested with HRP-streptavidin-conjugated secondary antibody at room temperature for 30 min. DAB and hematoxylin staining were used to develop the expression of TFE3 in tissues. The research protocol conducted was approved by the ethics committee of Shandong University on 26 January 2016 (protocol number: A9180112).

### EATR assay

The principle of EATR is as follows: when ER-phagy is not triggered, the tandem fluorescent proteins are stable in the cytoplasm, When ER-phagy occurs with lysosomes engulfing these two fluorescent proteins mediated by RAMP4, the green fluorescence will quench by the instability of GFP in acid lysosomes so as to leave red fluorescence from mCherry^22^.

### Luciferase report assay

TFE3 overexpression plasmid, pGL3 basic empty, E-Syt1 overexpression plasmid, E-Syt1 mutated plasmid, Syt7 overexpression plasmid, Syt7 mutated plasmid, FTH1 overexpression plasmid, FTH1 mutated plasmid were constructed by Genepharma (Shanghai, China). 293 T cells (8 × 10^4^ cells per well) were seeded into 24 well culture plate with 500 µl DMEM medium containing 10% FBS and cultured at 37 °C. On the second day, each group was transfected with indicated plasmids respectively. by transfection reagent Lipofectamine 3000 Reagent (Thermo Fisher). Then 48 h later, the double luciferase reporter assay system (Promega, Beijing, China) was performed by manufacturer’s instructions.

### Correlation analysis

The preprocessed and standardized expression value matrix data (series_matrix document) were downloaded from two GEO datasets and read by R package (3.6.1) limma package. For different probes mapped to the same gene, we take the mean value of different probes as the final expression value of the gene. The ENSEMBL ID of RNA -seq data of TCGA data (https://www.gencodegenes.org /) is reannotated with GTF annotation file in the instrument to obtain the corresponding gene symbol. The expression profiles of TFE3 and E-Syt1, Syt7, and FTH1 were extracted. Based on Pearson algorithm, nine expression value correlation graphs were drawn through R-package ggplot2.

### Transmission electron microscopy

Cells transfected with Syt7 or clinical tumor specimens (storage at −80 °C) were fixed with 2.5% glutaraldehyde overnight at 4 °C. After fixation with 1% osmium tetroxide for 1 h, we dehydrated all the samples with different concentrations of ethanol. Araldite was the embedding reagent. Ultrathin sections of (50 nm) were stained by uranyl 16 acetate and lead citrate. A cm-120 electron microscope (TECNAI 17 sprite biotwin, Fei, Thermo Scientific) was used to scan the slices.

### Quantitative PCR (qPCR)

RNeasy Mini Kit (Qiagen, Hilden, Germany) was used to extract total RNA. from tissues or cells. Reverse transcription of total RNA was processed by kit instructions from kit (Clontech, Mountain View CA). Quantitative PCR reactions were processed by Fast qPCR Mix SYBR Green Master Mix (TaKaRa, RR430S).

FTH1 forward, 5′-CAACAGTGCTTGGACGGAAC-3′

FTH1 reverse, 5′-GAGTCCTGGTGGTAGTTCTGG-3′

CTSB forward, 5′-CTCTTTCCATCCCCTGTCGG-3′

CTSB reverse, 5′-TAGCCACCATTACAGCCGTC-3′

Syt7 forward, 5′-GTCAGCCTTAGCGTCACTGT-3′

Syt7 reverse, 5′-GACTCATCGTGGGGTGTCTG-3′

E-Syt1 forward, 5′-GGTTGCTGGTGCTGATACCT-3′

E-Syt1 reverse, 5′-GGCAGGTAGCTCTCGATGAC-3′

Lamp1 forward, 5′-CAACACGTTACAGCGTCCAG-3′

Lamp1 reverse, 5′-CCTGGGTGCCACTAACACAT-3′

GAPDH forward, 5′-A AGCTCACTGGCATGGCCTT-3′

GAPDH reverse, 5′-C TCTCTTCCTCTTGTGCTCTTG-3′.

### Nuclear/cytoplasmic protein fractionation

Nuclear and cytoplasmic fractionation was performed according to The Kit protocols from Abcam (ab113474). After been centrifuged, the nuclear and the cytosolic fraction were collected respectively. Equal volumes of the nuclear and cytoplasmic lysates were tested by Western blot.

### Western blot and Co-immunoprecipitation

After lysing and extracting the total cell protein with RIPA buffer (Beyotime, Shanghai, China), we used BCA Protein Assay Kit (Beyotime, Shanghai, China) to determine the protein concentration. The protein samples were separated by 12% sodium dodecyl sulphate polyacrylamide gels electrophoresis (SDS-PAGE) (30 µg per lane) and then transferred onto the PVDF membrane (Millipore, Billerica, MA, USA). In total 5% skimmed milk in TBST (0.1% Tween-20 in PBS) was used to block the membrane for 1 h at room temperature, all the primary antibodies needed were used to incubate the membrane overnight at 4 °C. After washing 3 times by TBST, the bands were then incubated in secondary antibody for 1 h at room temperature. The enhanced chemiluminescence (ECL) reaction was detected by ChemiDoc XRS + system (Bio-Rad, Hercules, CA). Immunoblot image intensities were tested by ImageJ software. Cells collected from 10 cm dishes were prepared for immunoprecipitation and performed according to the Kit’ protocols from TaKaRa (635721).

### Live-cell image

After transfected with indicated plasmid, 786 O/OE-SR cells (5 × 10^^4^) were seeded in FluoroDish cell culture dishes (Shengyou Hangzhou China). After the culture medium was removed, post-transfected cells were washed with preheated PBS and counter stained with Hoechst 33342 for 10 min followed by washing in preheated PBS and incubated in preheated culture medium (Life technologies). Then we took the real-time imagines by confocal microscopy (Carl Zeiss Germany). Similarly for ER tracker (Serve Life Science, Shanghai China) staining, after 100 μl working solution incubated with indicated cells for 10 min, post transfected cells were washed with preheated PBS and stained with Hoechst 33342 for 10 min followed by washing in preheated PBS and incubated in preheated culture medium (Life technologies).

### Detection of intracellular Ca^2+^ concentration

After cells received indicated treatment, they were seeded in 6 well plates (5 × 10^5^ well for 14 h. Then cells were incubated with Fluo-3-AM(0.5 µm/well) at 37 °C for 10 min. Washing 3 times with PBS. The cells were then, and Ca^2+^ concentration was measured by flow cytometry analysis (BD Biosciences).

### Subcutaneous xenograft model and lung metastasis model

The female BALB/c nude mice (3 weeks of age) were purchased in batches from Beijing VitalRiver Laboratory Animal Technology Co., Ltd. All animal work procedures were approved by the Ethics Committee of the Shandong University Qilu Hospital. The 786 O cells stably expressing TFE3 were randomly injected subcutaneously into six nude mice (5 × 106 cells in 200 μl PBS, *n* = 3/group) at 4 weeks of age. Tumor volume was daily measured. Calculate formula of tumor Volume: (large diameter) × (small diameter)^2^/2. When the tumor volume approached about 200 mm^3^, the six tumors bearing mice were randomly assigned to two groups. Randomly one group received sunitinib treatment by tail injection (15 mg/kg/day) for 40 days and the other group received equivalent DMSO by tail injection for 24 days as P1 generation. One tumor from each group was cut into small pieces (2 × 2 mm) and implanted subsequently to the P2 generation of new nude mice (*n* = 3/group/generation). When the tumor volume approached about 200mm^3^, continuous sunitinib treatment (32 days) and DMSO treatment (24 days) were processed in P2 generation mouse. The establishment method of P3 generation is the same as above. The biggest tumor from each group in the P3 generation was selected for primary cell extraction and culture. The primary cells received DMSO treatment is named 786 O/OE and the primary cells received sunitinib treatment is named 786 O/OE-SR.

The pro-metastatic activities of Syt7 were tested by the mouse cancer lung metastasis model. 786 O/OE-SR cells were infected with lentivirus containing sh-Syt7 plasmid or empty vector plasmid as control and each group contained 5 nude mice. Infected cells were selected with 2 μg/ml puromycin for 1week. Syt7 knockdown cells and control cells (2 × 10^5^) were suspended in 200 μL of PBS and implanted by tail vein injection. Randomly each group of mice were treated with sunitinib(10 mg/kg/day) or DMSO for 45 days. The mice lungs metastasis was assessed by counting the number of nodules by staining the lung slides with Hematoxylin-eosin (H&E) staining.

### Statistical analysis

The data of three independent experiments were presented as means ± the standard deviation (sd). Student’s t-test was performed statistical comparisons by using of the results were done using GraphPad Prism 5 software. *P* value < 0.05 is considered as statistically significant and are indicated as follows: *P < 0.05; **P < 0.01; ***P < 0.001.

## Result

### Lysosomal pathway was reactively activated in mRCC after acquired sunitinib resistance

**Fig. 1:**
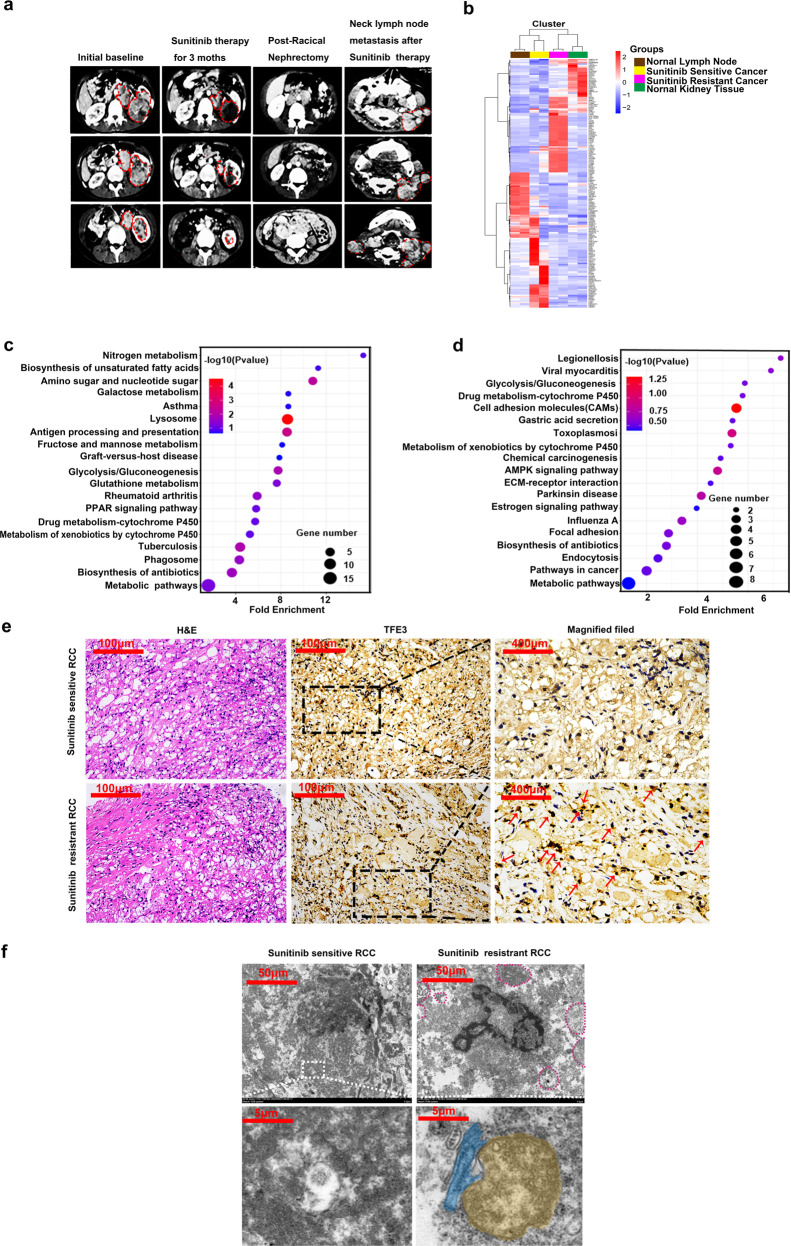
Translocation of TFE3 promoted metastasis of sunitinib-resistant RCC by mediating lysosome synthesis. **a** CT scans of different disease stages from a ccRCC patient: sunitinib sensitive stage, one month after radical nephrectomy, new metastatic lesions occurred in left lymph node by 4 years’ sunitinib treatment (from left to right). **b** Heat map of TMT proteomics from cancer tissues from different disease stages. **c** GO functional annotation analyzed the up-regulated proteins and the most differentially expressed proteins were enriched in the lysosome. **d** GO functional annotation analyzed the down-regulated proteins and the most differentially expressed proteins were enriched in CAMS, ECM receptor interaction, and focal adhesion. **e** Representative images of hematoxylin-eosin (H&E) staining and TFE3 staining by immunohistochemical (IHC) in sunitinib sensitive tissues and sunitinib resistant tissues. Scale bar, 100 μm and 400 μm. **f** Representative TEM images of lysosome (red dotted circle) in sunitinib sensitive tissues and sunitinib resistant tissues Scale bar 50 μm. Higher magnification insets showed the autophagosome (yellow) was engulfing the ER fragment (blue) in sunitinib-resistant tissues compared with sunitinib-sensitive tissues. Scale bar 5 μm.

A 24-year-old female diagnosed with mRCC using enhanced computed tomography (CT) scan in 2011 (Fig. 1a). In response to sunitinib therapy for 3 months, the tumor size had decreased significantly as shown in CT scan (Fig. 1a). We performed radical nephrectomy of the left kidney for this patient, and the pathological report was ccRCC (pT4N1M1) (Fig. 1e). This patient received postoperative maintenance with sunitinib, and regular follows-ups were performed to monitor the progress of the disease. After sunitinib therapy for 4 years, this case developed left cervical lymph node metastasis (Fig. 1a). We firmly believed that the tumor samples from the left kidney are sunitinib sensitive, and respectively the samples from lymph nodes are sunitinib resistant. We applied TMT Proteomics to analysis the sunitinib sensitive samples and paired lymph metastasis samples. Two sites were randomly selected for each lesion. The heat map analysis was illustrated in Fig. 1b. We analyzed the survival of the proteins with the most obvious difference by the TCGA database (Supplementary Fig. 1). Then bubble chart showed that lysosomal proteins were the most significantly up-regulated protein in metastasis sample compared to sunitinib sensitive tumor sample (Fig. 1c). Correspondingly, the down-expressed proteins were enriched in extracellular matrix, such as cell adhesion molecules (CAMs) and ECM-receptor interaction proteins (Fig. 1d). TFE3 was strongly expressed in the nucleus in the metastatic RCC tissue, and diffused in the cytoplasm in sunitinib sensitive tissue which theoretically consistent with the fact that sunitinib promoted TFE3 translocated into the nucleus by inhibiting VEGF-mTOR signaling pathway (Fig. 1e). There were more and bigger lysosomes in metastasis RCC samples than in sunitinib sensitive RCC samples (Fig. 1f). Interestingly, we surprisingly found abundant ER-phagy phenomena by transmission electron microscope (TEM) (Fig. 1f).

### TFE3 induced acquired sunitinib resistance and metastasis in RCC

**Fig. 2:**
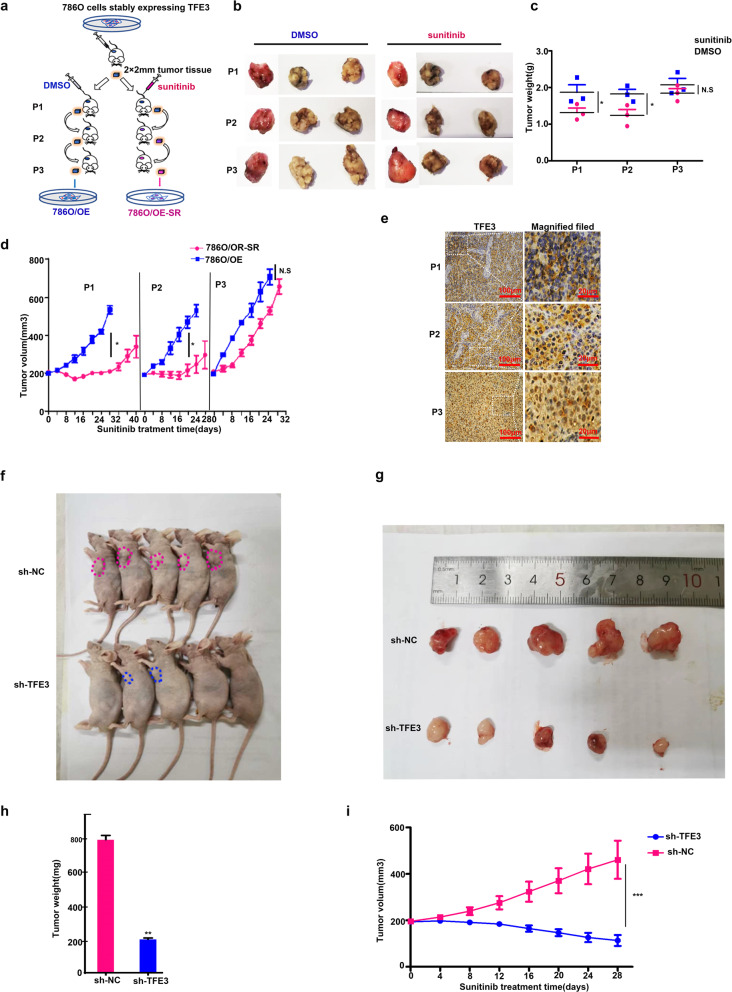
TFE3 is the core regulator of sunitinib resistance in vivo. **a** Describe the process of establishing two cell lines. Subcutaneous tumorigenesis in mice using 786 O cell lines stably expressing TFE3. Control group received DMSO (blue) treatment and experimental group received sunitinib (yellow) treatment. After three generations of treatment, the primary cells were extracted named 786 O/OE and 786 O/OE-SR correspondingly. **b**, **c** Weight of tumor xenografts in mice of every generation (two-tailed Student’s *t* test and two ways ANOVA, *P < 0.05, NS: not significant). **d** Tumor volume of every generation (two-tailed Student’s *t* test and two ways ANOVA, *P < 0.05, NS: not significant). **e** Representative images of IHC staining TFE3 in P1, P2, P3 xenografts Scale bar, 100 μm, indicated region 20 μm. **f**, **g** BALB/c nude mice were implanted with 786 O/OE-SR cells which were transfected with lentivirus containing sh-TFE3 or sh-empty vector (*n* = 5 per group) and all mice received sunitinib (15 mg/kg/day) treatment for 30 days. Representative photos of tumors were illustrated. **h**, **i** Tumor volumes and weights were lower in mice with TFE3 knockdown xenotransplant. **P < 0.01, ***P < 0.001.

In order to confirm the hypothesis that sunitinib can induce metastasis of RCC after drug resistance, we first established two cell line models named 786 O/OE and 786 O/OE-SR via in vivo experiments (Fig. 2a). By measuring the tumor volume and weight, we can see that the P1 and the P2 xenograft tumors had a fair response to sunitinib, and the P3 xenografts have already showed stable sunitinib resistance and the tumor volume slightly increased with the effect of the drug (Fig. 2b–d). IHC assay showed that in P1 and P2 generation, TFE3 was diffusely distributed in the cytoplasm, but in P3 generation, TFE3 was strongly expressed in the nucleus (Fig. 2e). TFE3 knockdown in 786 O/OE-SR cells significantly reduced the growth of tumor xenografts and can reverse the resistance to sunitinib in mice models (Fig. 2f–i)

### TFE3 is a core regulator of RCC metastasis induced by long-term sunitinib treatment in vitro

**Fig. 3:**
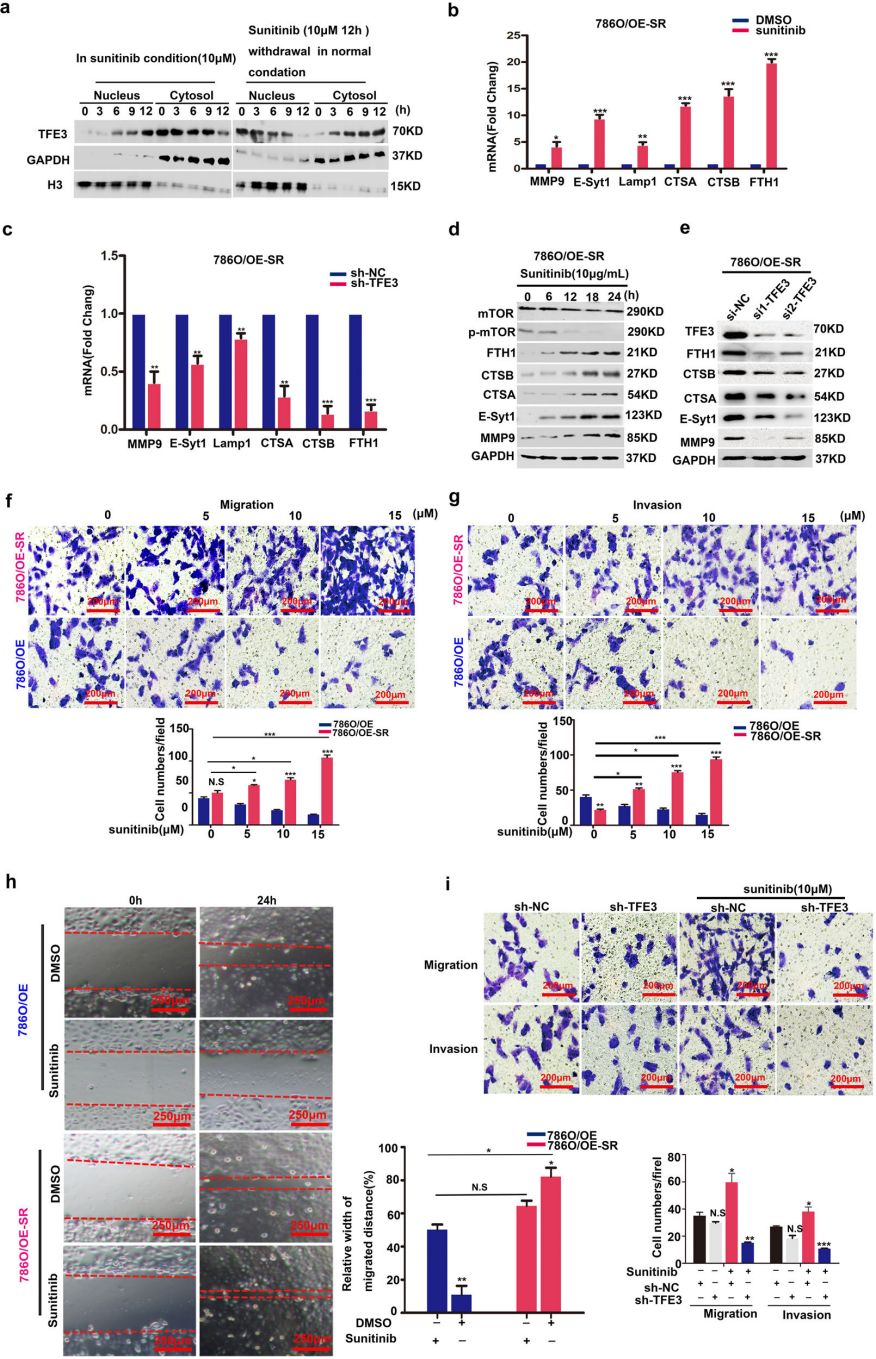
Sunitinib stimulated the metastasis of 786O/OE-SR cells by activating TFE3 translocation. **a** Western blot showed that expression and distribution of TFE3 was dynamic between the nucleus and the cytoplasm. GAPDH and Histone served as specific internal reference markers for cytoplasm and nuclei respectively. **b** The target genes of TFE3 were verified in786O/OE-SR cells treated with DMSO or sunitinib (10 μM) for 12 h. Data are shown as means ± SD. *P < 0.05, **P < 0.01, ***P < 0.001, NS: not significant. Data were obtained from three independent experiments. **c** After knocking down of TFE3, qPCR verified the target gene of TFE3 in 786 O/OE-SR cells treated with DMSO, sunitinib (10 μM) for 12 h. **P < 0.01, ***P < 0.001. Data are shown as means ± SD (*n* = 3). **d** Proteins downstream of TFE3 in 786 O/OE-SR cell line were upregulated with the increase in sunitinib concentration. **e** After knocking down of TFE3, 786 O/OE-SR were treated with sunitinib (10 μM) for 12 h cells and proteins downstream of TFE3 were down regulated. **f** Transwell assay to assess migration after 12 h incubation of 786 O/OE-SR with different concentration of sunitinib compared with 786 O/OE cells. Scale bar, 200 μm, ***P < 0.001, **P < 0.01, *P < 0.05, NS: not significant. Data are shown as means ± SD (*n* = 3). **g** Transwell assay to assess invasion after 12 h incubation of 786 O/OE-SR with different concentration of sunitinib compared with 786 O/OE cells. Scale bar, 200 μm. **P < 0.01, ***P < 0.001. Data are shown as means ± SD (*n* = 3). **h** Wound healing assays for 786 O/OE and 786 O/OE-SR cells with DMSO, sunitinib (10 μM) for 24 h. Scale bar, 250 μm. *P < 0.05, **P < 0.01, NS: not significant. Data are shown as means ± SD (*n* = 3). **i** Rescue experiments showed that knockout of TFE3 could reverse the effect of sunitinib on tumor metastasis in 786 O/OE-SR cells. Scale bar, 200 μm, *P < 0.05, **P < 0.01, NS: not significant. Data are shown as means ± SD (*n* = 3).

786 O/OE-SR cells showed stronger sunitinib resistance than 786 O/OE cells and 786 O cells (Supplementary Fig. 2a). TFE3 is restrained in the cytosol under normal conditions and rapidly translocated into the nucleus when in stress conditions^21^. WB experiments confirmed that the basal amount of TFE3 in the nucleus of 786 O/OE-SR cells was much more abundant than that in 786 O/OE cells (Supplementary Fig. 2b). Under normal conditions, TFE3 is restrained in cytosol, but rapidly translocates to the nucleus when cells suffer medium stress condition^21^. WB assay confirmed that the subcellular distribution of TFE3 mediated by sunitinib was a dynamic process. The expression of TFE3 in the nucleus increased in sunitinib condition. After drug withdrawal, TFE3 gradually transferred back to cytoplasm (Fig. 3a). From the TMT Proteomics data, we found that Ferritin and E-Syt1 are dramatically upregulated in mRCC tissue. By WB and qPCR assay, we confirmed that some targets filtered out from proteomics were the downstream proteins of TFE3 in 786 O/OE-SR cells (Fig. 3b–e). We also compared these targets at the mRNA and protein levels in 786 O/OE and 786 O/OE-SR cells (Supplementary Fig. 2c, d). There were more lysosomes in 786 O/OE-SR cells than in 786 O/OE cells (Supplementary Fig. 2e). In 786 O/OE-SR cells, with sunitinib concentration increased, the ability of migration and invasion were enhanced (Fig. 3f–h). Rescue experiments showed that sunitinib lost its ability to enhance tumor cell metastasis without TFE3 (Fig. 3i).

### As a direct regulatory target of TFE3, E-Syt1 acted upstream of FAM134B during ER-phagy

**Fig. 4:**
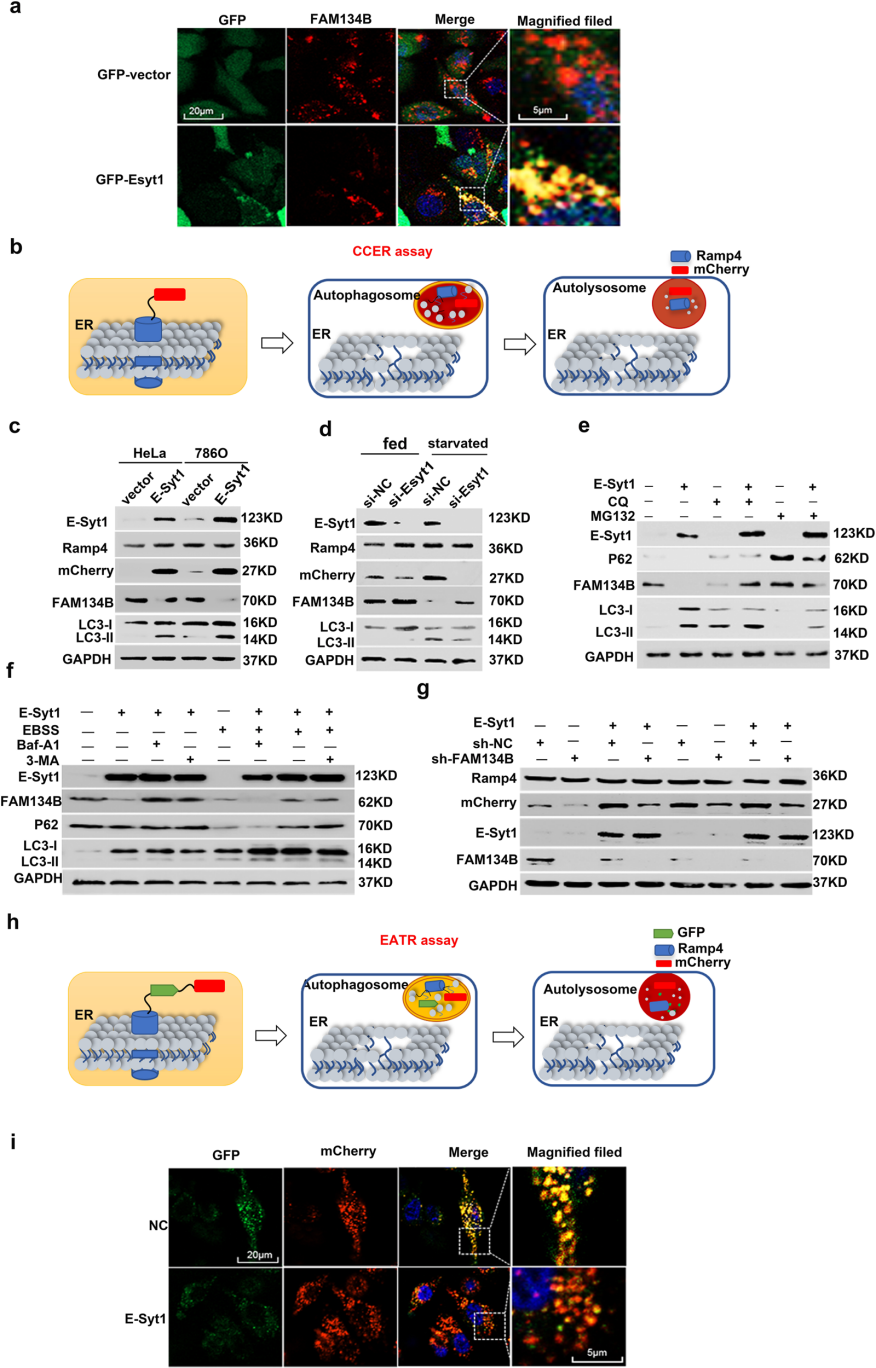
TFE3 can stimulate ER-phagy by promoting the expression of E-Syt1. **a** Confocal images of puncta formed by Esyt1-GFP and FAM134B-mCherry colocalized in 786 O/OE-SR cells. Scale bar 20 µm and 5 µm. **b** Diagram of CCER assay. When ER-phagy occurs, the lysozyme cleaved the link between mCherry and RAMP4, and mCherry became a small molecule with only 27 KDa which been detected by WB. **c** HeLa and 786 O/OE-SR cells stably expressing CCER system were transfected with E-Syt1 and Western blot tested ER-phagy degree. **d** 786 O/OE-SR cells were transfected with sh-Esyt1 plasmid or empty plasmid and starved for 12 h by EBSS before WB measurement. **e** Western blotting of 786 O/OE-SR cells transfected with Esyt1 and treated with MG132 (10 μM) or CQ (10 mM) for 12 h before harvest. **f** 786 O/OE-SR cells transfected with E-Syt1 were either in normal condition or in EBSS with or without 3-MA (500 nM), Baf -A1 (100 nM) for 12 h and harvested for WB. **g** 786 O/OE-SR cells were transfected with E-Syt1 and sh-FAM134B or one of the plasmids. Then cells were cultured in normal condition or in EBSS for 12 h. The result was shown by WB. **h** Schematic diagram of the EATR assay. When ER-phagy occurs, green fluorescence quenched in acid lysosome leaving only the red fluorescence excited by mCherry. **i** Confocal images of 786 O/OE-SR cells (stabling expressing EATR system) transfected with E-Syt1 fixed to visualize ER-phagy. Scale bar 20 µm and 5 µm.

David C. Rubinsztein et al. reported that TFEB upregulated autophagy by directly binding to the CLEAR element in the promoter region of Syt11^23^. We identified a homologous region of the CLEAR element in the promoter of E-Syt1, which was −1008bp away from the transcription start site (TSS) (Supplementary Fig. 3a). Correlation analysis showed that E-Syt1 was positively correlated with TFE3 by RNAseq data from TCGA and GEO databases (Supplementary Fig. 3b). Our dual-luciferase reporter assay proved that TFE3 could up-regulate E-Syt1 by directly binding to the CLEAR element (Supplementary Fig. 3c). FAM134B and RTN3L are two key receptors of ER-phagy and one of their common interacting partners is E-Syt1^24^. The co-localization of FAM134B and E-Syt1 was also observed by confocal microscopy (Fig. 4a). The distribution of E-Syt1 was different from other Esyts. E-Syt1 were widely distributed in cytoplasm, while E-Syt2 and E-Syt3 had a predominant PM-like localization^24^. It had reported that Esyts could recruit autophagy marker proteins at endoplasmic reticulum plasma membrane (ER-PM) contact site, thus promoting the assembly of autophagosomes^25^. RAMP4 is a subunit of the ER translocation complex and can be engulfed into lysosomal during ER-phagy^22^. We applied the mCherry cleavage from ER assay (CCER assay) to verify he regulation of ER-phagy by E-Syt1 (Fig. 4b)^22^. Western blotting assay showed that E-Syt1 acted upstream of FAM134B-mediated ER-phagy (Fig. 4c–g). We next used an ER autophagy tandem reporter assay (EATR assay) (Fig. 4h) to test the intensity of ER-phagy after overexpressing E-Syt1 in 786 O/OE-SR cells, and the results were consistent with those before (Fig. 4i).

### Lysosomal releasing enzymes directly degrade the ER into fragments for autophagosome sequestration

**Fig. 5:**
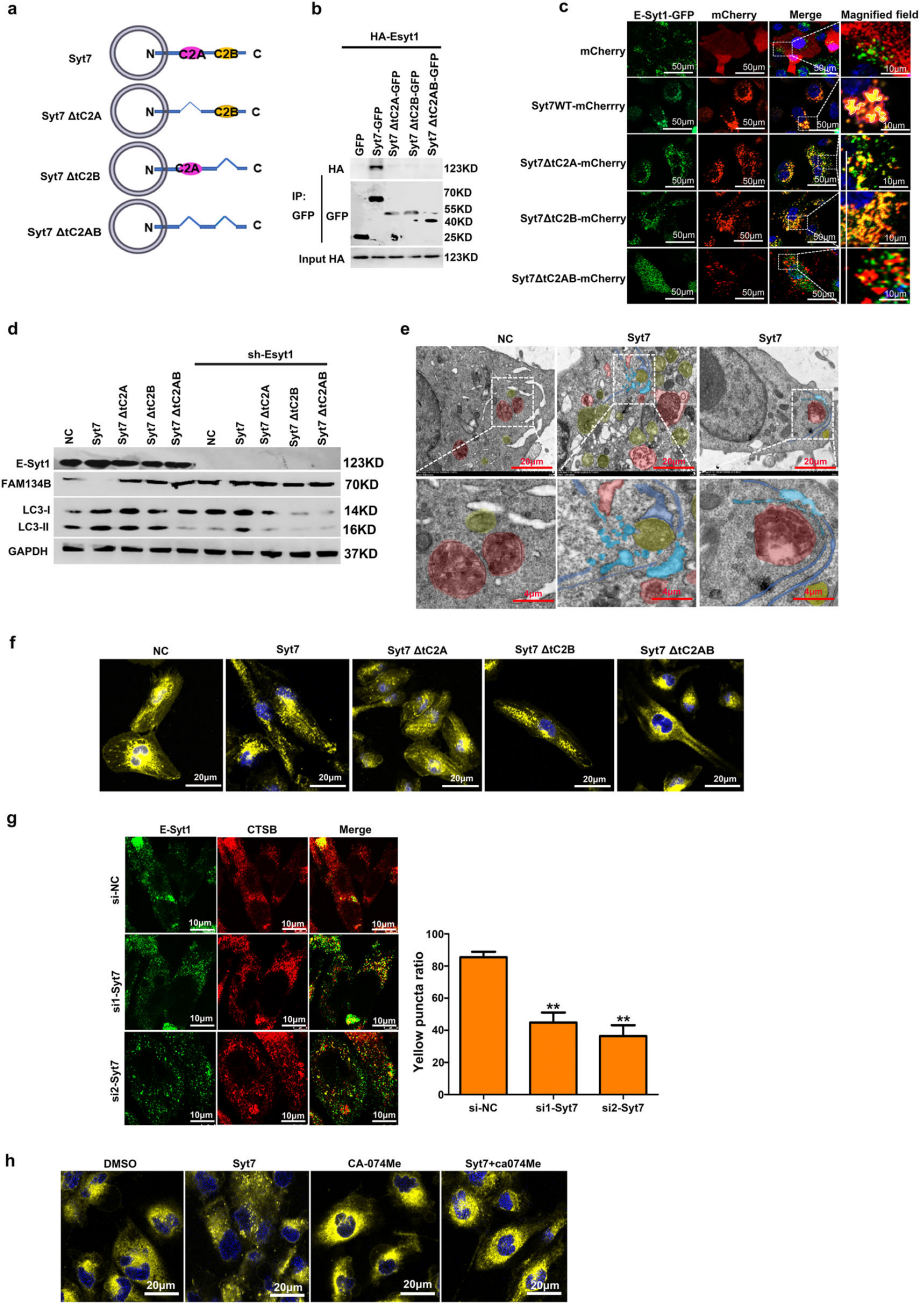
E-Syt1/Syt7 heterodimer mediated ER fragmentation directly by lysosomal enzyme. **a** Domain architecture of Syt7. **b** Co-IP of E-Syt1 and Syt7. 293 T cells transfected with HA-Esyt1 and indicated plasmids of Syt7 mutants. Syt7 mutant proteins were immunoprecipitated using anti-mCherry antibody and the precipitates were analyzed using anti-HA antibody. **c** 786 O/OE-SR cells stably expressing GFP-Esyt1 were infected with mCherry or mCherry-tagged mutants with Syt7 by lentivirus, and nucleus stained with Hoechst 33342. Representative confocal images were shown. Scale bar 50 µm. Enlargement images, scale bar 10 µm. **d** Indicated plasmids of Syt7 mutants were transfected in 786 O/OE-SR cells with or without knockdown of E-Syt1. Lysates of cells were tested by WB. **e** 786 O/OE-SR cells transfected with Syt7 or empty vector. Representative TEM images showed that in some ER-Lysosome contact sites, ER was fragmented and engulfed by autophagosomes. Red area depicted the lysosome, yellow area indicated autophagosome, blue area indicated ER, light blue area indicated ER fragment. Scale bar 20 µm. Enlargement images, scale bar 4 µm. **f** 786 O/OE-SR cells transfected with Syt7 or mutated vector were treated with ER Tracker (yellow) and nuclear dye Hoechst 33342 (blue). ER morphology was assessed by confocal microscopy. Scale bar 20 µm. Scale bar 20 µm. **g** Co-localization of Esyt1 and CTSB in 786 O/OE-SR cells after siRNA mediated knockdown of Syt7. Scale bar 10 µm. Thirty cells were randomly selected for each treatment. The number of binding sites between E-Syt1 and CTSB in each cell was counted and the experiment was repeated 3 times (**h**) 786 O/OE-SR cells transfected with Syt7 or empty vector were treated with CA-074Me (10 μM) or DMSO for 24 h and then cells were incubated with ER Tracker (yellow) and nuclear dye Hoechst 33342 (blue). ER morphology was assessed by confocal microscopy. Scale bar 20 µm.

In order to further explore the biological function of E-Syt1, we detected the proteins interacting with E-Syt1 in 786 O/OE-SR by mass spectrometry (MS), among which Synaptotagmin7(Syt7) attracted our interest. As a lysosomal membrane protein, Syt7 plays important role in lysosomal exocytosis, membrane resealing, and wound healing^26^. Analysis of the Syt7 promoter and the dual-luciferase reporter assay also proved that Syt7 was downstream of TFE3 (Supplementary Fig. 4a, b). We also detected proteins that interacted with Syt7 in 786 O, 786 O/OE, and 786 O/OE-SR cells by MS, and the results are shown in Figure. S4c. Syt7 contains two C2 domains involving in vesicle interaction and fusion (Fig. 5a). Syt7 can form heterodimer with Esyt1 through its domain C2 domains (Fig. 5b, c). The effect of Syt7 on the degradation of Fam134B depended on the existence of E-Syt1 (Fig. 5d). After overexpressing Syt7 in 786 O/OE-SR cells, we surprisingly found that in some ER-lysosome contact sites, the ER swelled and accompanied by many ER fragments in TEM imagines (Fig. 5e). ER fragmentation is an indispensable key step of the ER-phagy and it has been reported the oligomerization of ER-phagy receptors such as FAM134B and RTN3L were the core regulators of this process^24,27^. Confocal microscopy showed that the ER fragmentation induced by Syt7 extremely needed C2 domains (Fig. 5f). After siRNA silencing syt7, the diffuse distribution of CTSB decreased and the co-localization of CTSB with E-Syt1 decreased (Fig. 5g). Specific inhibition of lysosomal acid hydrolase CTSB by CA-074Me could rescue the effect of Syt7 on ER fragmentation (Fig. 5h).

### Ca^2+^ release triggered by ER-phagy promoted the lysosomal exocytosis via Syt7

**Fig. 6:**
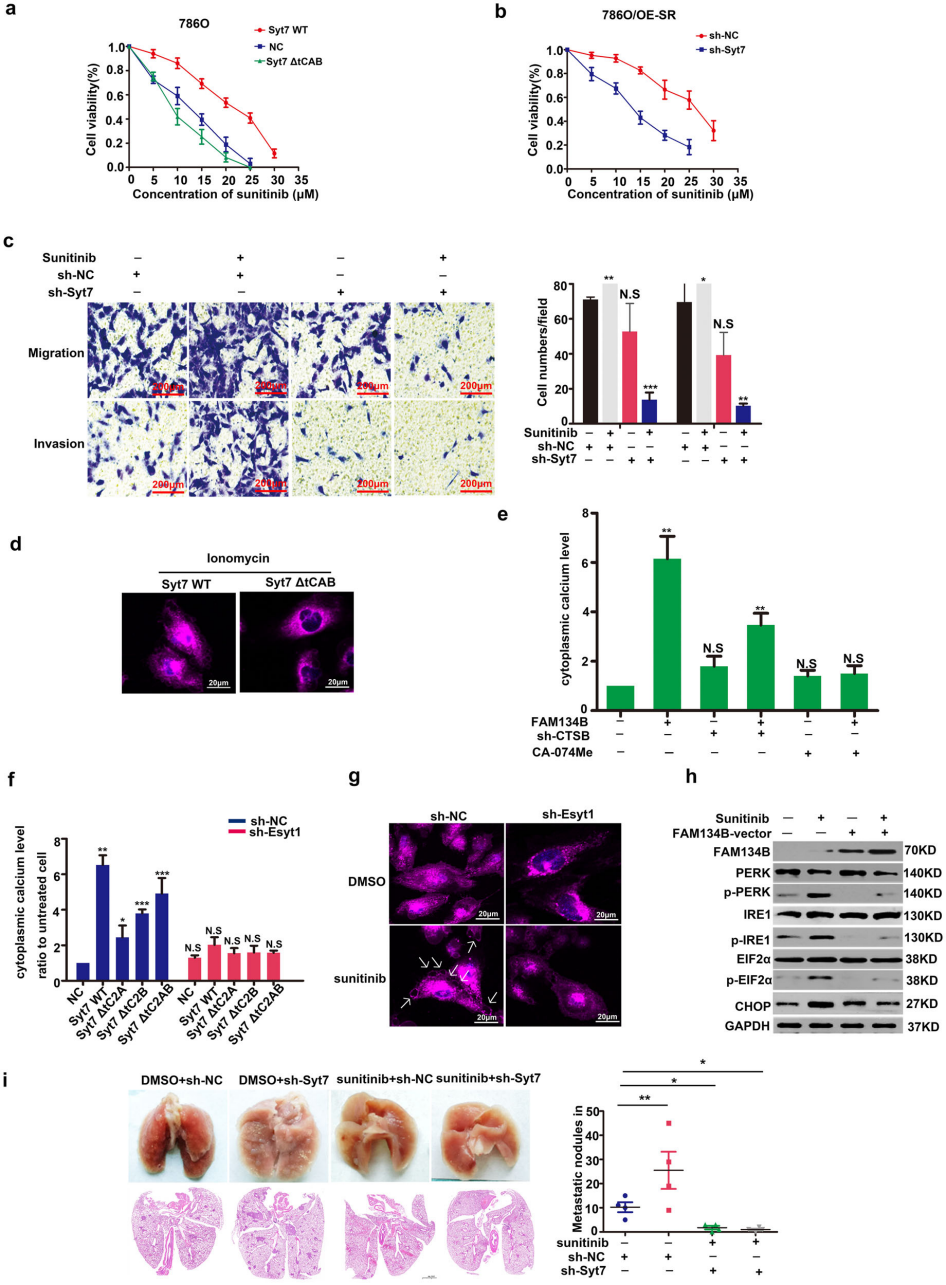
ER-phagy could promote Ca2^+^ release so as to induce Syt7 mediated lysosomal exocytosis and metastasis in 786O/OE-SR cells. **a** CCK8 showed the viability of 786 O cells or transfected with Syt7, Syt7ΔtC2AB with increasing concentration of sunitinib. Data were obtained from three independent experiments. **b** CCK8 showed the viability of 786 O/OE-SR cells or Syt7 knockdown 786 O/E-SR cells with increasing concentration of sunitinib. Data were obtained from three independent experiments. **c** Transwell assay showed that knockdown of Syt7 could reverse the effect of TFE3 on tumor metastasis in 786 O/OE-SR cells in the condition of sunitinib (10 µM). Scale bar, 200 μm ***P < 0.001, **P < 0.01, *P < 0.05. Data were obtained from three independent experiments. **d** Confocal images of vesicle exocytosis. 786 O/OE-SR cells transfected with mCherry-Syt7 or mCherry-Syt7 ΔtC2AB were incubated with ionomycin (2 µM) 2 h. White arrows indicate lysosome. Scale bar 20 µm. **e** 786 O/OE-SR cells transfected with FAM134B were treated with or without CA-074Me, sh-CTSB. Cells were incubated with 2 µm Fluo-3-AM at for 30 min and then flow cytometry was used to test the relative Ca2^+^ concentration. The results represent the mean ± SD. **P < 0.01, NS: not significant. Data were obtained from three independent experiments. **f** 786 O/OE-SR cells with or without E-Syt1 knockdown were transfected with indicated plasmids of Syt7 mutants. Cells were incubated with 2 µM Fluo-3-AM for 30 min and then flow cytometry was used to test the relative Ca2^+^ concentration. Results represent the mean ± SD. *P < 0.05, **P < 0.01, ***P < 0.001, NS: not significant. Data were obtained from three independent experiments. **g** Confocal images of vesicle exocytosis. 786 O/OE-SR cells stably expressing mCherry-Syt7 were treated with DMSO or sunitinib (10 µM) for 12 h and were transfected with sh-Esyt1 or sh-NC. White arrows indicate lysosome. Scale bar 20 µm. **h** 786 O/OE-SR cells transfected with or without FAM134B-vector were incubated with sunitinib (10 µM) or DMSO for 12 h, WB tested ER stress-related markers. **i** Representative lungs and corresponding H&E sections from different cohorts were shown. mice injected with Syt7 knockdown 786 O/OE-SR cells showed significantly reduced metastasis incidence in the condition of sunitinib treatment. *n* = 5, *p < 0.05, **P < 0.01.

**Fig. 7:**
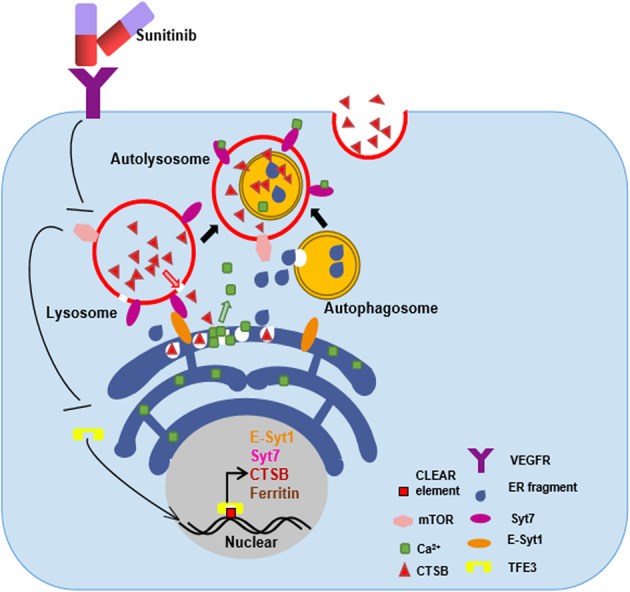
The schematic illustrates the phenomena and mechanisms described in this paper. Sunitinib stimulated TFE3 to enter the nucleus, which promoted the transcription of a series of genes, and finally promoted lysosome exocytosis through ER-phagy and calcium release.

C2 domains were associated with the resistance to sunitinib in 786 O cells (Fig. 6a). Knockdown of Syt7 temporarily by sh-RNA could reverse the resistance to sunitinib in 786 O/OE-SR cells (Fig. 6b). Transwell assay showed that knocking down Syt7 could reverse the promotion of metastasis by sunitinib in 786 O/OE-SR (Fig. 6c). Ionomycin is a Ca2^+^ vehicle by carrying extracellular Ca^2+^ into the cytoplasm so as to trigger lysosomal exocytosis. C2 domains function as Ca^2+^ sensor and can indeed trigger lysosomal exocytosis in 786 O/OE-SR cells (Fig. 6d). By measuring the intracellular calcium concentration, overexpress FAM134B could directly increase intracellular Ca^2+^ concentration, and inhibition of CTSB can significantly reduce this process (Fig. 6e). We found that the heterodimer formed by Syt7 and E-Syt1 was essential for Ca^2+^ release (Fig. 6f). Sunitinib did induce lysosome exocytosis via Syt7, and this process was at least partially mediated by knockdown of E-Syt1 in our results by confocal microscopy (Fig. 6g). Because the WB results showed that the ER-phagy mediated by FAM134B could reduce the degree of ER stress, we believe that the Ca^2+^ release was not caused by ER stress (Fig. 6h). This at least suggested that ER-phagy could alleviate ER stress in drug-resistant cells. In vivo experiments also showed that knocking down Syt7 could reverse the promotion of metastasis by sunitinib in (Fig. 6i). In other words, TFE3-Esyt1/Syt7 axis induced Ca^2+^ release by directly degrading and fragmenting ER.We draw a modle proposed on how TFE3-Esyt1/Syt7 axis regulate ER-phagy Ca^2+^ release and lysosomal exocytosis in Fig. 7.

## Discussion

Frequent inactivation of VHL gene and activation of HIF-VEGF pathway are the main molecular biological characteristics of renal cell carcinoma, which are also the theoretical basis of antiangiogenic drug therapy. RTK inhibitors targeting VEGFR such as sunitinib and sorafenib are the first line molecular-targeted drugs for the treatment of mRCC^28^. Acquired resistance to RTK inhibitors and tumor metastasis are the main causes of treatment failure. However, in some tumor models, RTK inhibitor treatment increased the incidence of distant metastasis in mice^29–34^. Most of these reports focus on breast cancer and pancreatic cancer. Yang et al. reported that TFEB can mediate immune evasion and metastasis of RCC by inducing PD-L1 expression^35^. The MiT family has four major genes (TFE3, TFEB, TFEC, and MiTF). At present, most of the studies on TFE3 and cancer focus on Xp11.2 translocation renal cell carcinoma (tRCC) but not ccRCC, a special type of RCC characterized by gene fusions involving TFE3 or TFEB. Molecular signatures from proteomics gave us new insight into the metastasis mechanism of RCC after acquired sunitinib resistance. MiT family members are the most significant transcription factors by directly upregulating lysosome biosynthesis. We found that only TFE3 was upregulated in proteomic data without any other MiT family members. The VEGF/VEGFR2 pathways have three downstream: the JAK/STAT, MEK/ERK, and PI3K/AKT pathways. It has been demonstrated that 3-kinase (PI3K) is induced by VEGF/VEGFR2 can phosphorylate phosphoinositide 3-kinase (PI3K) and AKT, both of which augment mammalian target of rapamycin (mTOR) activation. mTOR complex 1 (mTORC1) can respond to the nutrients level change by regulating the intracellular distribution of TFE3^20,21,36^. In fed condition, TFE3 at serine 321 (Ser321) residue of TFE3 is phosphorylated by phosphorylated mTOR as a result of recruiting cytosolic chaperone 14-3-3 to bind to TFE3. 14-3-3 can limit the translocation of TFE3 to the nucleus^21,37,38^. When mTORC1 is inhibited, TFE3 will be translocated into the nucleus rapidly without the restriction of 14-3-3^36^. The above evidence is sufficient to indicates that sunitinib treatment can induce tumor metastasis through TFE3. Our proteomic results clearly indicated that lysosomal enzymes promote tumor metastasis by releasing them to the ECM. In other words, sunitinib has become the initiator of metastasis of innately drug-resistant RCC.

A large amount of ER-phagy was observed in metastatic tumor samples by TEM. The function of ER-phagy in cancer is unclear. Carmine settembre et al. reported that MiT/TFE factors can trigger ER-phagy by directly transcriptional regulation of FAM134B^39^. We found that E-Syt1 is downstream of TFE3 and acted upstream of FAM134B during ER-phagy. The main function of E-Syt1 is ER-PM tethering^40^. In the initial stage of ER-phagy, FAM134B recruited LC3 to ER and meanwhile the FAM134B mediated ER fragmentation. The growing phagophores assembled by LC3 eventually engulf the ER fragments and become autophagosome^41^. Membrane tethering mediated FAM134B oligomers is so far the clearest mechanism of ER fragmentation^27^. We found that Esyt1 and Syt7 (lysosome membrane protein) could form heterodimer so as to mediates the contact between lysosome and ER and lysosomal enzymes were directly released to the nearby ER in cytoplasm, resulting in swelling and fragmentation of ER. We also observed that the ER fragmentation and nearby mature autophagosome engulfing ER fragments were two relatively independent processes. We did not observe the phenomenon described in the literature: phagophore encapsulates ER fragments during growth and extension, and finally completely closed to become autophagosome^41^. Our findings at least provide a new perspective for the study of ER fragmentation. We further explored the relationship between ER-phagy and metastasis of drug-resistant tumors cells and found that Syt7 was a very important linker between these biological phenomena. ER fragmentation mediated by heterodimerization between Syt7 and E-Syt1 resulted in the releasing of calcium ions from ER to the cytoplasm. As a calcium sensor on lysosomal membrane, Syt7 can sense the increase of calcium ion concentration in the cytoplasm and trigger lysosome exocytosis. We speculate that exocytosis of lysosome can not only eliminate the isolated sunitinib in lysosome, but also degrade the ECM to promote metastasis of tumor cells.

There is a kind of clinical subtype of RCC which will develop rapid disease progression after TKI therapy^42^, the case we studied is not of this type. The patient received a satisfactory progression-free survival (PFS) by sunitinib treatment and experienced obvious tumor shrinkage. Sunitinib is still the best choice for this case. Therefore, it is urgent to monitor the resistance of sunitinib in RCC during treatment. Based on proteomic data. Our proteomics results also confirmed Marija Petkovic’s hypotheses that mTOR pathway inhibitors will also result in changes in iron and ferritin level in mRCC^42^. The relationship between TFE3 and ferritin needs further exploration.

TFE3 is highly expressed in RCC tissue. Although the expression of TFE3 or TFEB in RCC has no statistical significance for prognosis, it does not mean that there is no significance under the effect of targeted drugs. This study suggests that it is necessary to monitor the sensitivity of the tumor to sunitinib in some mRCC patients with high expression of TFE3 or TFEB.

## Supplementary information

Supplementary figure legend

Supplementary figure 1

Supplementary figure 2

Supplementary figure 3

Supplementary figure 4
