# Supplementary material for: Sunitinib treatment promotes metastasis of drug-resistant renal cell carcinoma via TFE3 signaling pathway
Source: Cell Death Dis. 2021 Feb 26;12(2):220. doi: 10.1038/s41419-021-03511-3 (PMC7910457; doi:10.1038/s41419-021-03511-3)
Supplement: Supplementary file 1 — Supplementary figure legend [file 41419_2021_3511_MOESM1_ESM.docx]

**Supplementary Figure legend**

**Supplementary Figure 1.** (a) Survival analysis of genes involved in lysosomal enzyme, ECM, and iron metabolism.

**Supplementary Figure 2.** (a) CCK8 showed the viability of 786O cells, 786O/OE cells, 786O/OE-SR cells with increasing concentration of sunitinib. Data were obtained from three independent experiments. (b) Western blot showed that expression and distribution of TFE3 between the nucleus and the cytoplasm in 786O/OE cells and 786O/OE-SR cells. (c) The target genes of TFE3 were verified in 786O cells after transfected with or without TFE3 for 48h. *P<0.05, **P<0.01, ***P<0.001. Data were obtained from three independent experiments. (d) Western blot and qPCR compared targets in 786O/OE cells and 786O/OE -SR cells. (e) Representative TEM images of 786O/OE and 786O/OE-SR cells. Regions of interest were lysosome exocytosis and were outlined with white dashed lines and magnified to the right. Lysosome numbers were quantitatively analyzed in 786O/OE and 786O/OE-SR cells. *P<0.05.

**Supplementary Figure 3.** (a) The schematic illustrates the promoter region of E-Syt1. (b) Correlation analysis of E-Syt1 and TFE3 from TCGA and GEO databases. (c) Double luciferase reporter assay verified that TFE3 can promote E-Syt1 transcription via the CLEAR element.

**Supplementary Figure 4.** (a) The schematic illustrates the promoter region of Syt7. (b) Double luciferase reporter assay verified that TFE3 can promote Syt7 transcription via the first CLEAR element. (d) The proteins interacting with Syt7 were identified by Co-IP and mass spectrometry, and their functions were annotated.
